# In-Clinic versus Hybrid Cancer Rehabilitation Service Delivery during the COVID-19 Pandemic: An Outcome Comparison Study

**DOI:** 10.3390/curroncol30100644

**Published:** 2023-09-29

**Authors:** Kelley C. Wood, Smith Giri, Tiffany D. Kendig, Mackenzi Pergolotti

**Affiliations:** 1 ReVital Cancer Rehabilitation, Select Medical, Mechanicsburg, PA 17055, USA; tkendig@selectmedical.com (T.D.K.); mpergolotti@selectmedical.com (M.P.); 2 Institute for Cancer Outcomes and Survivorship, University of Alabama at Birmingham, Birmingham, AL 35233, USA; smithgiri@uabmc.edu; 3 Department of Occupational Therapy, University of North Carolina at Chapel Hill, Chapel Hill, NC 27599, USA

**Keywords:** telerehabilitation, quality of life, outpatients, patient-reported outcome measures, neoplasms, cancer survivors, health care quality, access and evaluation, rehabilitation, physical therapy specialty, occupational therapy

## Abstract

Diminished health-related quality of life (HRQOL) is common among cancer survivors but often amendable to rehabilitation. However, few access real-world rehabilitation services. Hybrid delivery modes (using a combination of in-clinic and synchronous telehealth visits) became popular during the COVID-19 pandemic and offer a promising solution to improve access beyond the pandemic. However, it is unclear if hybrid delivery has the same impact on patient-reported outcomes and experiences as standard, in-clinic-only delivery. To fill this gap, we performed a retrospective, observational, comparative outcomes study of real-world electronic medical record (EMR) data collected by a national outpatient rehabilitation provider in 2020–2021. Of the cases meeting the inclusion criteria (*N* = 2611), 60 were seen to via hybrid delivery. The outcomes evaluated pre and post-rehabilitation included PROMIS^®^ global physical health (GPH), global mental health (GMH), physical function (PF), and the ability to participate in social roles and activities (SRA). The patient experience outcomes included the Net Promoter Survey (NPS^®^) and the Select Medical Patient-Reported Experience Measure (SM-PREM). A linear and logistic regression was used to examine the between-group differences in the PROMIS and SM-PREM scores while controlling for covariates. The hybrid and in-clinic-only cases improved similarly in all PROMIS outcomes (all *p* < 0.05). The association between the delivery mode and the likelihood of achieving the minimal important change in the PROMIS outcomes was non-significant (all *p* > 0.05). No between-group differences were observed in the NPS or SM-PREM scores (all *p* > 0.05). Although more research is needed, this real-world evidence suggests that hybrid rehabilitation care may be equally beneficial for and acceptable to cancer survivors and supports calls to expand access to and reimbursement for telerehabilitation.

## 1. Introduction

Ignited by the COVID-19 pandemic, the use of telerehabilitation or rehabilitation services delivered using synchronous communication technology [1] has exploded across the United States to maintain continuity of care and access to outpatient rehabilitation services. In cancer care specifically, telerehabilitation holds exciting promise to solve a long-standing clinical challenge: poor access to cancer-specialized rehabilitation services to manage survivors’ functional and health-related quality of life (HRQOL) needs [2,3]. Of the 18.1 million cancer survivors living in the United States as of January 2022 [4], 60–75% have at least one unmet need related to function or HRQOL that may be amenable to rehabilitation [5,6,7,8]. When unaddressed, these needs can increase, persist, and potentially compound in the presence of the late and lasting effects of cancer treatment. For example, a recent prospective cohort study including over 55,000 cancer and non-cancer controls showed that survivors of the most common cancer types report significantly poorer functioning than age-matched controls 5 years following diagnosis [9]. Despite the high prevalence of survivors with unmet needs, accessing cancer rehabilitation services remains a significant challenge [8,10], with transportation to the clinic cited as one of many barriers that impede access to care [11].

Telerehabilitation could decrease barriers to access by delivering services to patients when and where they need them. Research trials performed prior to the COVID pandemic suggest telerehabilitation is safe and feasible [12,13,14,15,16,17,18,19,20], acceptable to survivors and clinicians [12,13,14,16,18], and associated with improved functional and/or psychosocial outcomes [12,13,14,15,18,19,20,21]. When coupled with the high rates of smartphone ownership in the United States (89% of adults) [22], these findings suggest telerehabilitation now, more than ever, could be leveraged as a flexible and practical way to expand access to cancer-specialized rehabilitation services [21,23,24]. However, early evidence suggests that although telerehabilitation may be feasible to maintain continuity of care and improve patient-reported outcomes measures [20], telerehabilitation alone may not be sufficient to meet cancer survivors’ needs and could introduce new barriers to care [19,20,25]. Survivors attending telerehabilitation in these studies reported diminished feelings of peer and therapist support, difficulty with digital technology, and that home approaches were heavily reliant on self-motivation [19,20,25]. When asked, outpatient therapists delivering telerehabilitation have also reported challenges, including technology, the ability to instruct and monitor patients during exercise, and the ability to provide educational resources [25]. For these reasons, many patients and therapists report they would prefer a flexible blend or hybrid delivery mode of telerehabilitation and in-clinic care [16,26,27].

Hybrid delivery methods could be used to maximize access to and the impact of cancer rehabilitation care beyond the COVID-19 pandemic. The existing research supports that offering a mix of in-clinic and at-home telerehabilitation based on the patient’s needs may minimize a patient’s barriers to attending in-clinic appointments (e.g., transportation, time, appointment burden) while maintaining the rigor of high-specialized therapeutic interventions and hands-on care [14,28,29]. Yet, there is little evidence to indicate if the patient-reported HRQOL and patient experience outcomes of hybrid services are comparable to in-clinic-only services. Comparing these outcomes can help to inform payor and policy decisions regarding ongoing support for telerehabilitation as a mode of service delivery beyond the public health emergency and pandemic. The aim of this study was to compare the HRQOL and patient experience outcomes of cancer survivors who attended hybrid and in-clinic-only services delivered as standard practice.

## 2. Materials and Methods

This is a retrospective, observational study of real-world EMR data collected by a national outpatient rehabilitation provider from 1 January 2020 to 31 December 2021. We report the study methods following the Professional Society for Health Economics and Outcomes Research (ISPOR) checklist for retrospective database studies [30].

### 2.1. Cancer Rehabilitation Service Delivery

Cancer survivors attended outpatient PT or OT appointments provided by a single-institution provider of outpatient cancer-specialized rehabilitation services in the United States (ReVital Cancer Rehabilitation, Select Medical). PT or OT services were delivered in a community-based outpatient rehabilitation clinic (i.e., in-clinic only) or via a combination of in-clinic and synchronous telerehabilitation appointments (i.e., hybrid). Within the institution, telerehabilitation was offered during the COVID pandemic as a new mode of service delivery for patients to continue treatment while keeping them and employees safe. Telerehabilitation sessions were offered based on patient preferences, state-based COVID restrictions and practice acts, and therapist clinical judgment and discretion as standard of practice. Telerehabilitation sessions were conducted using the Doxy.me platform. Rehabilitation interventions were provided by cancer-specialized therapists following typical practice. Therapists providing telerehabilitation received additional training on telerehabilitation best practices, including patient consent and safety, technical skills, remote assessment, and the collection of outcome measures. The content and frequency of rehabilitation interventions were determined by ir therapists based on the individual patient’s needs and goals. All services were billed through insurance for skilled therapy needs per typical practice. 

### 2.2. Case Identification and Data Extraction

Cases that met the following criteria were included in the study: (1) seen for an initial PT or OT evaluation within the study period (January 2020–December 2021), (2) reported cancer diagnosis (identified via ICD10 code[s] applied by the treating therapist), and (3) had patient-reported outcome measures (PROM) at an initial evaluation (pre) and at discharge (post). An honest broker used these criteria to identify eligible cases in the electronic medical record (EMR) to create de-identified dataset for the analysis. The available rehabilitation characteristics included delivery mode (hybrid or in-clinic-only), the number of sessions attended, duration (weeks from initial evaluation to discharge or last visit date), discipline (PT or OT), and clinic location via United States region. The available patient characteristics included age at the start of rehabilitation, sex, cancer type via ICD 10 code, and payer type (federally funded, private, other). For the between-groups analysis and regression modeling, we categorized cancer type into the following four groups based on similarities in cancer biology, treatment, and baseline PROM scores: (1) breast, (2) gastrointestinal, genitourinary, colorectal, or gynecologic; (3) heme or lymphoid; and (4) other (head and neck; lung or respiratory; endocrine or neuroendocrine; brain; or central nervous system, and skin). 

### 2.3. Outcome Measures

Outcome measures included three PROMs and two PREMs.

#### 2.3.1. Patient-Reported Outcome Measures

Developed by the National Institutes of Health (NIH), PROMIS^®^ instruments are validated with state-of-the-science methods to be psychometrically sound and are freely available for research and clinical use [31]. The following PROMIS short forms were evaluated: Global Health (10 items, scored as two domains: Global Physical Health [GPH] and Global Mental Health [GMH]), Physical Function (4 items, PF), and Ability to Participate in Social Roles and Activities (4 items, SRA). In addition to their well-validated psychometric properties, these PROMIS measures provide a global perspective of HRQOL, and these measures have been previously shown to be associated with important cancer outcomes including frailty, morbidity, and mortality [32,33]. In addition, growing research demonstrates that these measures are appropriate to monitor patient health as part of routine rehabilitation evaluation and to quantify the impact of cancer rehabilitation interventions on HRQOL [34,35]. PROMIS instruments are scored on a T-score scale (mean: 50, standard deviation: 10) following the standardized guidelines for each instrument. A higher T-score indicates more of the domain measured, in this case, superior global physical or mental health, physical functioning, and the ability to participate in social roles and activities. Two points represents the minimal important change (MIC) [36]. 

#### 2.3.2. Patient Experience Measures

Patient experience measures include the Net Promoter Survey (1-item, NPS) [37] and the Select Medical-Patient-Reported Experience Measure (11-items, SM-PREM©) [38]. The NPS is widely used across healthcare organizations and research to understand patient experience via their likelihood to recommend the services they receive [37]. The NPS asks “How likely are you to recommend (this facility) to family/friends?”; patients respond on an 11-point Likert scale (0 [not at all likely] to 10 [extremely likely]). Based on their likelihood to recommend it, patients are categorized into one of three groups: “promoter” (9–10), “passive” (7–8), or “detractor” (≤6) [37]. At an aggregate level, the NPS score is calculated by subtracting the proportion of detractors from the proportion of promoters (NPS score = [% promoters] − [% detractors]); the possible range is −100 to 100. An accepted NPS score benchmark for the outpatient rehabilitation industry is 84 [39]; however, within the institution, 90.1 [38] and 91.4 [40] are the accepted benchmarks for orthopedic and cancer rehabilitation services, respectively, and therefore these were used as the benchmark in this study. The NPS was administered automatically by the EMR system after an initial evaluation, 6 weeks after the evaluation, and following discharge. For this study we extracted the rating closest to the date of discharge.

The SM-PREM© was developed and validated by the institution, demonstrating strong internal consistency to measure patient experience in a study of over 89,000 patients attending orthopedic outpatient PT and OT [38]. The SM-PREM includes 11 items in three categories: “facility and front desk staff”, “your clinical care”, and “your overall impression”. Patients rate each item on a 5-point Likert scale from 0 (strongly disagree) to 5 (strongly agree). The overall score is calculated by dividing the sum of all item responses by 11. The SM-PREM is only administered electronically following a patient’s discharge from rehabilitation [38].

### 2.4. Statistical Analysis

To examine the between-group differences in patient and rehabilitation treatment characteristics, we used independent samples *t*-tests or Mann–Whitney U tests for continuous variables and Pearson chi-squared (Χ^2^) or Fisher’s exact test tests for categorical variables. For each PROMIS outcome, we used a paired samples *t*-test to examine the within-group change from initial evaluation to discharge, then used a multiple linear regression to model to evaluate the effect of the delivery mode (hybrid vs. in-clinic only) on the T-score change while controlling for covariates (age, sex, cancer type, U.S. region, visits, and therapy discipline [PT vs. OT]). In each regression model, we evaluated the change in scores instead of the post-score value to account for differences in the baseline scores. We used a binary logistic regression to model the impact of the delivery mode on the likelihood of achieving the MIC on each PROMIS outcome while controlling for the same covariates. The level of significance was set to α = 0.05 for all hypothesis tests. All analyses were performed using IBM SPSS Statistics (Version 27).

## 3. Results

The cancer rehabilitation cases (N = 2611) were 60.80 ± 12.9 years old (range: 19–95), mostly female (80.3%), and most had a diagnosis of breast cancer (64.5%). Most cases participated in PT (87.0%) and had private (50.2%) health insurance. Sixty cases were seen using a hybrid delivery mode. Hybrid cases attended approximately 29% of their appointments using telerehabilitation (IQR: 7% to 64%) rather than in-clinic appointments. Descriptively, when compared to those who attended in-clinic-only rehabilitation, the hybrid cases attended more PT/OT visits (median: 18 vs. 10, *p* < 0.001) over a longer period (median: 14.79 vs. 9.00 weeks, *p* < 0.001) and were more likely to be in the Northeastern region of the United States (48.3%) than other regions (*p* < 0.001). There were no other between-group differences in the patient or rehabilitation characteristics (Table 1). 

### 3.1. PROMIS^®^ HRQOL Measures

**Figure 1:**
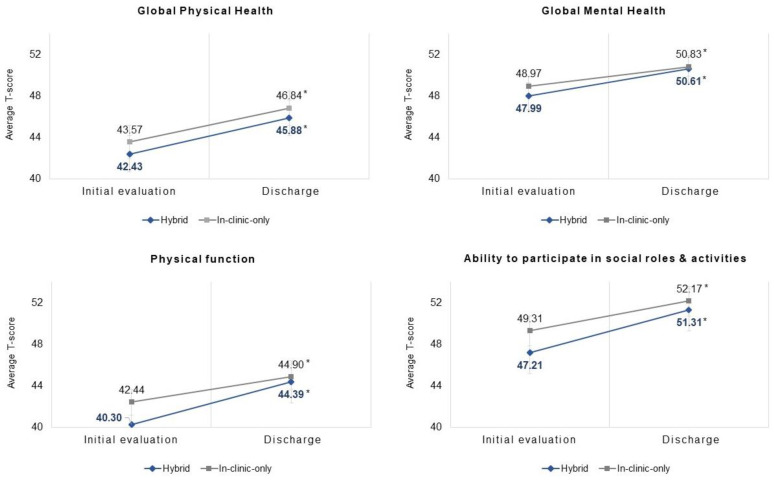
PROMIS^®^ HRQOL outcomes, hybrid vs. in-clinic-only cancer rehabilitation. * Significant within-group improvement in T-score from initial evaluation to discharge, *p* < 0.05. Between-group differences in T-score improvement from initial evaluation to discharge were non-significant for each measure: GPH (*p* = 0.846), GMH (*p* = 0.415), PF (*p* = 0.118), and SRA (*p* = 0.287).

At the initial evaluation, the PROMIS T-scores were similar between the hybrid and in-clinic-only cases (all *p* > 0.05). From the initial evaluation to discharge, the hybrid cases (N = 60) improved significantly in each PROMIS measure: GPH (3.46 ± 7.44, *p* = 0.001), GMH (2.62 ± 7.63, *p* = 0.011), PF (4.09 ± 7.45, *p* < 0.001), and SRA (4.10 ± 8.37, *p* = 0.001). The in-clinic-only cases (N = 2551) also improved significantly in each PROMIS measure: GPH (3.26 ± 7.21, *p* < 0.001), GMH (1.85 ± 7.08, *p* < 0.001), PF (2.46 ± 7.24, *p* < 0.001), and SRA (2.86 ± 8.93, *p* < 0.001). The average T-scores at each time point are plotted in Figure 1.

When compared to the in-clinic-only cases, the hybrid cases improved similarly in each PROMIS measure: GPH (*p* = 0.846), GMH (*p* = 0.415), PF (*p* = 0.118), and SRA (*p* = 0.287). However, the hybrid cases exceeded the MIC in each PROMIS measure, whereas the in-clinic-only cases achieved the MIC on three of four PROMIS measures (GPH, PF, and SRA). When controlling for covariates, the delivery mode (hybrid vs. in-clinic-only) was not associated with a superior improvement on any PROMIS measure (GPH [*p* = 0.62], GMH [*p* = 0.54], PF [*p* = 0.47], and SRA [*p* = 0.83]; Table 2), nor was it a significant predictor of achieving the MIC (GPH [*p* = 0.36], GMH [*p* = 0.65], PF [*p* = 0.36], and SRA [*p* = 0.62]; Table 3).

### 3.2. Patient-Reported Experience Measures (PREM)

The NPS response rate was 65% among the hybrid cases (n = 39) and 47% among the in-clinic-only cases (n = 1186). The SM-PREM response rate was 53% among the hybrid cases (n = 20) and 16% among the in-clinic-only cases (n = 384). The overall NPS score was 87.2 (87.2% promoters, 0% detractors) for the hybrid cases compared to 92.3 for in-the clinic-only cases (94.2% promoters, 1.9% detractors). The median SM-PREM score was 5.00 (IQR: 4.32 to 5.00) for the hybrid cases compared to 5.00 (IQR: 4.57 to 5.00) for the in-clinic-only group. When compared between the hybrid and in-clinic care cases, there was no difference in the NPS rating (0–10 scale, Χ^2^ = 12.28, *p* = 0.20), the proportion of NPS promoters (Χ^2^ = 6.08, *p* = 0.44), the SM-PREM item scores (all *p* > 0.05), or the SM-PREM overall scores (*p* = 0.642).

## 4. Discussion

In this study, we found similar improvements in HRQOL and positive patient experience outcomes among the survivors who received either hybrid or in-clinic care. This suggests that both modes of delivery are beneficial and lends support for telerehabilitation as an ongoing mode of service delivery. 

To our knowledge, this is the first comparative outcomes study of a national sample of patients who attended cancer rehabilitation, evaluating the patient-reported impact and experience of hybrid-delivered PT and OT services in comparison to the outcomes of standard, in-clinic-only services. This study adds to the existing literature by using in-clinic-only cases treated during the same time as a comparison group. The results of this study align with previous, smaller studies investigating the effectiveness of real-world tele- [41] or hybrid-delivered rehabilitation for cancer survivors [42] and support previous conclusions that hybrid services may offer a “best of both worlds” care delivery model—allowing for flexible yet highly specialized and effective care [16,26,27].

In this study, the hybrid rehabilitation cases improved significantly in HRQOL outcomes, including physical health, mental health, physical function, and the ability to participate in social roles and activities. The improvements in HRQOL observed in this study align with previous studies, including real-world cancer rehabilitation patients in the United States [42], United Kingdom [20], and Australia [16]. For example, Helm and colleagues (2023) reported similar improvements in exercise and self-care efficacy outcomes in a survey study of 32 American women with breast-cancer-related lymphedema who participated in hybrid or in-clinic-only rehabilitation during the COVID-19 pandemic [42]. Similarly, in a prospective observational study of survivors participating in pre-treatment telerehabilitation by Wu and colleagues (2021) [20], the authors reported significant improvements in self-perceived health and fatigue. Dennett and colleagues (2021) also observed significant improvements in outcomes, including physical activity level, in their single-group process evaluation study of 123 survivors who received telerehabilitation during the pandemic. However, these studies are limited by small sample sizes and recall bias and do not compare their outcomes with traditional in-clinic care [16,20]. Therefore, the findings of this study are the best available evidence to understand the impact of the outcomes of hybrid versus in-clinic-only services delivered in the United States health care system. Notably, the changes in HRQOL observed in the present study were similar to or greater than those observed in our previous non-randomized study of 185 survivors attending in-clinic-only care provided by the same institution in 2019 [35]. 

This study, to our knowledge, is the first to examine validated patient experience measures captured during routine hybrid cancer rehabilitation care. Previous studies have relied on retrospective surveys [42] and interviews or focus group methods [16,20] which are often limited by response and recall bias. In contrast, the patient experience in this study was collected using validated measures that were administered and completed anonymously within or immediately following care. Therefore, although patient experience data were available for only 65% cases in this study, our findings may be more representative than previous studies. In contrast to the existing research literature, in which authors report mixed effects, patient experience did not differ between hybrid and in-clinic-only cases in this study. In a previous study of 205 patients who received telerehabilitation or home rehabilitation following a total knee replacement, Moffet and colleagues (2016) reported that patient satisfaction was high for both groups and not significantly different [43]. Yet, in a cross-sectional study including over 1000 patients with sports medicine needs treated during COVID-19 and the immediate post-pandemic period, Kim and colleagues (2022) observed significant differences in patient experience scores in the proportion of promoters on the NPS (telehealth: 75% vs. in-clinic: 89% promoters, *p* = 0.008), suggesting that patient experience was poorer for telehealth patients [44]. The authors suggested that a lack of connection and ‘hands-on’ interventions between therapists and patients negatively influenced patient experience for the telehealth group. In the present study, 87% of hybrid participants and 92% of in-clinic-only participants who completed the NPS were promoters. Although more research is needed to understand the optimal delivery of hybrid services, the higher NPS score observed in the present study may indicate that hybrid patients were less negatively impacted by the factors reported by Kim and colleagues (2022) [44]. 

Previous authors have suggested that hybrid models may enhance a cancer survivor’s ability to maintain appointments by reducing barriers to rehabilitation, including transportation restrictions, inclement weather, illness, anxiety, and depression, and time constrictions due to work or other responsibilities [28,45,46]. In our study, the survivors in the hybrid group attended more visits over a longer period, suggesting that having the option of telerehabilitation may have enhanced the accessibility of services by minimizing the barriers. Furthermore, the findings of our study suggest that hybrid care models may be especially appropriate and beneficial for individuals reporting poorer mental health. Although both groups were discharged above the Unites States general population normative T-score of 50, indicating that their mental health was “on par” with the general United States population, those who attended hybrid care in this study had poorer (but not significantly different) GMH scores at the initial evaluation. In addition, the average improvement between hybrid and in-clinic care on all outcomes was non-significant when controlling for covariates. The findings of this study suggest that hybrid telerehabilitation may be an effective alternative to in-person care for patients, especially those who experience barriers to attending all visits through in-clinic-only care. The assessment of the duration and length of rehabilitation intervention in this study adds valuable information for the practical aspects of hybrid delivery. Further research should continue to evaluate the optimal delivery methods of cancer rehabilitation services, especially for those who experience barriers to participating in in-clinic-only care. 

### 4.1. Limitations

As with any retrospective study of real-world data, there are inherent limitations such as the reliance on existing data collected for purposes other than research and limited access to confounding variables that could potentially impact outcomes (e.g., patient’s co-morbid conditions, cancer stage, grade, and treatments received). In addition, the hybrid group was relatively small compared to the in-clinic-only group, potentially limiting the detection of any between-group differences, especially in the categorical patient experience outcomes. Furthermore, because the participants and therapists selected the care delivery modes (as opposed to being randomized), bias may also influence the findings of this study. That being said, the hybrid group was powered above 80% to detect a significant within-group difference in the primary outcome (GPH).

The limited ability to control for all the potential confounding variables is another important limitation of this study. We included the available covariates in the main effects analyses, although additional variables could be investigated in future studies, including diagnosis stage and treatment type and status [46], patient digital literacy and confidence [19,20], and the therapist’s experience with telerehabilitation [16,26]. We recommend future studies to capture these additional covariates to better understand how they may, or may not, influence the effectiveness of rehabilitation interventions. Finally, the intent of this study was to evaluate patient-reported outcomes and experiences. Future studies including performance-based measures could enhance the understanding of the comparative outcomes achieved via hybrid and in-clinic-only rehabilitation interventions. 

### 4.2. Implications for Clinical Practice and Future Directions

Our findings demonstrating significant improvements of similar magnitude for both modes of delivery can help support the case to preserve reimbursement for telerehabilitation long beyond the COVID-19 pandemic. By making specialized, skilled PT and OT services accessible when and where survivors need them, hybrid delivery could meet the unmet health and well-being needs of cancer survivors. As the technology used in healthcare rapidly changes, understanding the impact of technology on clinical workflow, processes, and institutions will be critical to ensuring sustainable delivery modes and promoting equitable access to new modes of care [24]. For example, as the use of telerehabilitation exploded at the beginning of the pandemic, the use of telerehabilitation also sharply declined once clinics were re-opened [25]. This steep increase and decrease in usage is likely not representative of patient preference but due to a lack of time to fully flush out a sustainable organizational process. Hopefully, the window of opportunity to determine how to successfully integrate telerehabilitation into clinical practice is still open, even as severe cuts to insurance coverage are looming. As Saaei and Klappa (2021) advised, and we agree, pretending that telerehabilitation and digital health is going away could hold the field of rehabilitation back. Instead, we need to embrace the technology and platforms needed to increase quality (and equitable) access to care while maintaining the therapeutic relationship between patients and therapists [25].

## 5. Conclusions

In this study, the survivors who attended hybrid-delivered outpatient cancer PT and OT rehabilitation services improved significantly in terms of HRQOL outcomes and reported a highly positive patient experience. Furthermore, there were no differences in HRQOL improvement or patient experience for those who attended hybrid versus in-clinic-only care after controlling for covariates. Although more research is needed, these findings suggest that hybrid rehabilitation may be equally as beneficial and acceptable to patients as traditional in-clinic-only care and support calls to expand the access to and reimbursement for telerehabilitation services beyond the COVID-19 pandemic.

## Figures and Tables

**Table 1:** Patient and rehabilitation characteristics, hybrid versus vs. in-clinic-only cancer rehabilitation.

	Hybrid (*n* = 60)	In-Clinic Only (*n* = 2551)	Between-Group Comparison, *p*-Value
Age (Mean ± SD)	59.92 ± 13.08	60.82 ± 12.90	0.53
Sex (N, %)			0.63
Female	50, 83.3	2047, 80.2	
Male	10, 16.7	504, 19.8	
Cancer type (N, %)			0.40
Breast	34, 56.7	1270, 57.6	
Gastrointestinal, genitourinary, colorectal, or gynecologic	9, 15.0	334, 13.1	
Heme or lymphoid	8, 13.3	177, 6.9	
Other	7, 11.7	294, 11.5	
U.S. Region (N, %)			<0.001 *
Midwest	5, 8.3	294, 11.5	
Northeast	29, 48.3	456, 17.9	
South	13, 21.7	1173, 46.0	
Southeast	10, 16.7	466, 18.0	
West	3, 5.0	162, 6.4	
Payer type (N, %)			0.97
Federally funded	1223, 47.9	28, 46.7	
Private	1279, 50.1	31, 51.7	
Other	49, 1.9	1, 1.7	
Rehabilitation discipline (N, %)			0.80
Physical Therapy	53, 88.3	2219, 87.0	
Occupational Therapy	7, 11.7	332, 13.0	
Length of care, weeks (Median, IQR)	14.79 (10.18, 26.21)	9.00 (5.42, 14.14)	<0.001 *
Visits (Median, IQR)	18.00 (10.0, 23.5)	10.00 (6.00, 16.00)	<0.001 *

* Between-groups difference, *p* < 0.05. Between-groups comparisons completed using independent samples *t*-test or Mann-Whitney U test for continuous variables and Pearson chi-squared (Χ^2^) or Fisher’s exact test tests for categorical variables.

**Table 2:** Multiple linear regression model to examine the impact of delivery method on PROMIS^®^ T-score change.

Outcome Measure	Unadjusted Model		Adjusted Model *	
	β, 95% CI	*p*-Value	Β (95% CI)	*p*-Value
Global physical health	0.19 (−1.67, 2.06)	0.840	−0.17 (−2.05, 1.71)	0.857
Global mental health	0.76 (−1.07, 2.60)	0.415	0.73 (−1.14, 2.60)	0.445
Physical function	1.63 (−0.33, 3.58)	0.103	1.16 (−0.799, 3.13)	0.245
Ability to participate in social roles and activities	1.24 (−1.17, 3.65)	0.312	0.51 (−1.93, 2.95)	0.682

Comparator = in-clinic-only delivery. * Adjusted model covariates: age, sex, cancer type, U.S. region, payer type, therapy discipline (PT or OT), visits, and length of care.

**Table 3:** Binary logistic regression model to examine the impact of delivery method on odds of achieving the minimal important change (MIC) in PROMIS^®^ outcomes.

Outcome Measure	Unadjusted Model		Adjusted Model *	
	Odds Ratio, 95% CI	*p*-Value	Odds Ratio, 95% CI	*p*-Value
Global physical health	1.07 (0.63–1.8)	0.794	0.85 (0.49–1.49)	0.574
Global mental health	0.84 (0.50–1.41)	0.507	1.19 (0.69–2.04)	0.694
Physical function	1.52 (0.74–2.31)	0.130	1.45 (0.80–2.63)	0.215
Ability to participate in social roles and activities	0.91 (0.53–1.57)	0.739	1.03 (0.57–1.85)	0.931

Comparator = in-clinic-only delivery. * Adjusted model covariates: age, sex, cancer type, U.S. region, payer type, therapy discipline (PT or OT), visits, and length of care. Note: a within-groups change of two points on the T-score scale represents the MIC [36].

## Data Availability

Restrictions apply to the availability of these data. Data were obtained from Select Medical and are available from the authors with the permission of Select Medical.
